# The eIF3a translational control axis in the Wnt/β-catenin signaling pathway and colon tumorigenesis

**DOI:** 10.1016/j.canlet.2024.217303

**Published:** 2024-10-15

**Authors:** Zizheng Dong, Anuj Ojha, Lincoln Barlow, Liyun Luo, Jing-Yuan Liu, Jian-Ting Zhang

**Affiliations:** Department of Cell and Cancer Biology, The University of Toledo College of Medicine and Life Sciences, Toledo, OH, USA

## Abstract

Translational initiation in protein synthesis is an important regulatory step in gene expression and its dysregulation may result in diseases such as cancer. Translational control by eIF4E/4*E*-BP has been well studied and contributes to mTOR signaling in various biological processes. Here, we report a novel translational control axis in the Wnt/β-catenin signaling pathway in colon tumorigenesis by eIF3a, a Yin-Yang factor in tumorigenesis and prognosis. We show that eIF3a expression is upregulated in human colon cancer tissues, pre-cancerous adenoma polyps, and associates with β-catenin level and APC mutation in human samples, and that eIF3a overexpression transforms intestinal epithelial cells. We also show that eIF3a expression is regulated by the Wnt/β-catenin signaling pathway with an active TCF/LEF binding site in its promoter and that eIF3a knockdown inhibits APC mutation-induced spontaneous colon tumorigenesis in APC^min/+^ mice. Together, we conclude that eIF3a upregulation in colon cancer is due to APC mutation and it participates in colon tumorigenesis by adding a translational control axis in the Wnt/β-catenin signaling pathway and that it can serve as a potential target for colon cancer intervention.

## 1. Introduction

Translational control at the rate-limiting initiation step is a crucial regulatory mechanism in gene expression. Dysregulation of eukaryotic initiation factors (eIFs) or other factors in mRNA translation such as tRNAs in this process contributes to various abnormalities and diseases [1–4]. It has been well documented that dysregulation of eIF4E and its binding partner, 4EBP, promotes tumorigenesis [5–8]. Additionally, it is widely recognized that eIF4E and 4EBP play key roles in signal transduction through the rapamycin/mTOR pathway [9–12]. However, while eIF4E and 4EBP have been studied extensively in the context of tumorigenesis and mTOR signaling, other eIFs including eIF3a remain relatively underexplored, and their roles in translational control and signal transduction are still poorly understood.

eIF3a is a 170-kDa protein consisting of three putative motifs: the 91-amino acid PCI domain present in subunits of 26S Proteasome, COP9, and eIF3 complexes [13]; the 112-amino acid spectrin motif [14]; and a 10 amino-acid tandem repeat [15]. While eIF3a is considered a putative subunit of the eIF3 complex, it may not be essential for eIF3 function in pre-initiation complex formation [16]. For instance, eIF3a knockdown reduces global protein synthesis by only ~25% [17], although a later study indicated that it severely imparis translation initiation [18].

Interestingly, it has been shown that eIF3a knockdown increases translation of a subset of mRNAs including those encoding DNA repair proteins [19–22], which may act as tumor suppressors. This suggests that eIF3a may inhibit the synthesis of these proteins, potentially contributing to tumorigenesis. Recent PAR-CLIP studies have identified binding sites for eIF3a, along with eIF3b, eIF3d, and eIF3g, in the 5′-UTR sequences of mRNAs and these bindings appear to either inhibit or stimulate translation of the mRNAs [23]. These findings contrasts the prevailing veiw that most eIFs, such as eIF4E, generally enhance mRNA translation and protein synthesis, particularly for poorly translated mRNAs.

Elevated eIF3a expression has been observed in several human malignancies and is associated with better prognosis [20,24]. It has also been shown that eIF3a knockdown reverses malignant phenotype of cancer cells [17,25], yet paradoxically increases their resistance to DNA-damaging chemotherapeutics by upregulating synthesis of DNA damage repair proteins [19–22]. These findings suggest that eIF3a may be a “Yin-Yang” factor in cancer by regulating the synthesis of DNA damage repair proteins. However, it remains unknown what regulates eIF3a expression and how its upregulation contributes to both tumorigenesis and cancer cell sensitivity to chemotherapeutics.

In this study, we sought to address these questions using colon cancer as a model. We demonstrated that eIF3a functions as a proto-oncogene regulated by the Wnt/β-catenin signaling pathway. Mutations in the APC gene increase eIF3a expression by promoting its gene transcription, thereby driving tumorigenesis. Knocking down eIF3a expression disrupts APC mutation-induced intestinal and colon tumorigenesis. Thus, the Wnt/β-catenin pathway, like the mTOR pathway, contains a translational control axis mediated by eIF3a, which could serve as a novel therapeutic target in colon cancer.

## 2. Materials and methods

### 2.1. Materials

Antibodies against eIF3 subunits, including eIF3b (SC-137214), eIF3c (SC-74507), eIF3d (SC-271515), eIF3e (SC-74505), eIF3f (SC-390413), eIF3h (SC-271283), eIF3i (SC-374156) and eIF3j (SC-376651) were obtained from Santa Cruz Biotechnology (Santa Cruz, CA). The antibody against eIF3a (ABD) was generated inhouse and has been characterized previously [15]. Antibodies against eIF3g (PIPA526261), Pan-Ras antibody (MABS195), and GAPDH (Ab8245), as well as cell culture media and reagents were purchased from Invitrogen (Waltham, MA), EMD Millipore (Temecula, CA), and Abcam Inc. (Cambridge, MA), respectively. Antibodies against β-actin (A5316), rabbit anti-goat IgG antibodies (AP106P), and horseradish peroxidase-labeled goat anti-mouse IgG (A4416) and goat anti-rabbit IgG (A9169) were products of Sigma-Aldrich (St Louis, MO). All other reagents of molecular biology grade were from Sigma or Fisher Scientific (Chicago, IL).

### 2.2. Cell lines and transfection

The immortalized normal rat intestinal epithelial cell line RIE-1, as well as RIE-iH Ras cells harboring an inducible Ha-Ras^Val12 gene, were kindly provided by Dr. Hongmiao Sheng [26]. The human colorectal adenocarcinoma cell line CaCo-2 was obtained from ATCC (Manassas, VA) and reauthenticated using short tandem repeat (STR) profiling. RIE-1 and CaCo-2 cells were cultured in Dulbecco’s Modified Eagle Medium (DMEM) supplemented with 10% fetal bovine serum, 100 U/mL penicillin, and 100 μg/mL streptomycin. RIE-iH Ras cells were cultured in selective DMEM medium containing 400 μg/mL G418. For confluency studies, CaCo-2 cells were seeded in 60-mm dishes at a density of 1 × 10^6^ cells/dish and cultured for various durations [27], with the medium changed every two days.

To establish stable eIF3a-over-expression clone, RIE-1 cells were seeded in 100-mm dishes and transfected with either the pCβA/p170 construct or pCβA empty vector using Lipofectamine Plus reagent. Twenty-four hours after transfection, cells were selected using G418 [28]. Positive clones were identified using Western blot analysis of eIF3a expression. All stable clones were maintained in DMEM complete medium containing 300 μg/ml of G418.

### 2.3. eIF3a promoter-reporter construct engineering and luciferase reporter assay

Genomic DNA was extracted using the genomic DNA extraction kit (Qiagen) and used as templates to clone the full-length eIF3a promoter by PCR, with primers eIF3aProF and eIF3aProR (Supplemental Table S1). The full-length promoter was then used as a template to generate deletion mutant promoter sequence by PCR using primers (Table S1) eIF3aProF and eIF3aPromut 1 for ΔDS-TCF, CH3aF and eIF3aProR for ΔUS, and primers eIF3aPromut2 and eIF3aProR for ΔUS-TCF. All PCR products were cloned into the pGEM-T easy Vector (Promega). The promoter sequences were then released from the pGEM-T easy by digestion using *Hin*d III and *Nco*I, and subcloned into pGL3-Basic Vector with firefly luciferase reporter gene (Promega) at the *Hin*d III and *Nco*I sites, resulting in luciferase reporters driven by the eIF3a promoters. All constructs were confirmed by DNA sequencing.

Dual luciferase reporter assay was used to measure promoter activity as previously described [29]. Briefly, 1 × 10^5^ cells per well in a 12-well--plate were transfected with the firefly luciferase reporter constructs and the control pRL-TK, which encodes Renilla luciferase reporter to assess transfection efficiency, using Lipofectamine 3000 (Invitrogen). To evaluate the effect of APC or TCF on the eIF3a promoter activity, pcDNA3-APC, pcDNA3-TCF4, or pCDNA3.1 empty vector were co-transfected together with the reporter plasmids. For assessing the effect of β-catenin on eIF3a promoter activity, β-catenin siRNAs (Santa Cruz Biotechnology) or scramble control siRNA (Invitrogen) were co-transfected with the reporter plasmids.

Forty-eight hours post-transfection, cells were harvested for lysate preparation, and firefly and Renilla luciferase activity were measured using the Dual-Luciferase Reporter assay system (Promega) according to the manufacturer’s instructions. Firefly luciferase activity was normalized to that of Renilla luciferase and then to either the empty vector or scrambled siRNA control. Each transfection was performed in triplicate and repeated at least three times.

### 2.4. Tissue sample preparation and Western blot analysis

Frozen surgical specimens from 22 pairs of matched human colon cancer and adjacent normal tissues, as well as adenomas from 8 patients, were obtained from the IU Simon Comprehensive Cancer Center Tissue Procurement and Distribution Core. All tissues were verified by certified pathologist at the IU Simon Comprehensive Cancer Center. Preparation of human tissue samples for Western blot analysis were conducted as previously described [15,30,31]. Briefly, frozen human colon specimens were homogenized in cold TNN buffer (50 mM Tris-HCl, pH 7.4; 100 mM NaCl; 5 mM EDTA; 0.5% Nonidet P-40; 0.5 mM EGTA; 50 mM NaF, 1 mM Na_3_VO_3_, 200 μM phenylmethylsulfonyl fluoride [PMSF], 1 mM dithiothreitol [DTT]) and cleared of debris by centrifugation at 13,400g for 15 min. The supernatant was collected, and protein concentration was determined using Bradford reagent. Total lysate from cultured cells was prepared similarly.

Proteins from tissue or cell lysates were separated by SDS-PAGE and analyzed by Western blot analysis using antibody against eIF3a ABD (1:1000), eIF3b (1:200), eIF3i (1:200), eIF3g (1:600), GAPDH (1:20000), or β-actin (1:3000). Signal was detected using enhanced chemiluminescence (ECL).

### 2.5. Real-time RT-PCR analyses

Total RNA from cells or tissues were extracted using RNeasy Mini kit (Qiagen) according to the manufacturer’s instructions. Real-time RT-PCR was performed as previously described [32]. Briefly, 1 μg total RNA was reverse transcribed to synthesize cDNA using iScript^™^ cDNA Synthesis Kit (Bio-Rad). PCR was carried out on ABI Prism@7000 Sequence Detection System (Applied Biosystems) using SYBR Green detection, following manufacturer’s protocol. Primers used were eIF3aRTF and eIF3aRTR for eIF3a, and GAPDHF and GAPDHR for internal control GAPDH (Table S1).

The threshold cycle (Ct) of eIF3a was normalized to the Ct of GAPDH, and the experimental group was then normalized to the control group to calculate the relative RNA levels using the 2^−ΔΔCt^ formula. All experiments were performed in triplicate and repeated at least three times independently.

### 2.6. Alkaline phosphatase (ALP) and sucrase activity assays

Alkaline phosphatase and sucrase assays were performed as previously described [27]. Briefly, cells were harvested, washed twice in PBS, and lysed in Tris-mannitol buffer (2 mM Tris and 50 mM mannitol, pH7.1) by sonication. The lysates were cleared by centrifugation at 13, 400g for 15 min at 4 °C and then used for alkaline phosphatase and sucrase activity assays.

### 2.7. Anchorage-independent growth assay

Anchorage-independent growth in soft agar was performed as previously described [17]. Briefly, a base layer of 0.7% (wt/vol) Noble agar (Difco) in DMEM containing 10% FBS was prepared in 60-mm dishes, followed by the addition of a top layer containing 1000 cells in DMEM with 10% FBS and 0.4% (wt/vol) Noble agar. Cells were cultured for 14–21 days before colonies were stained with 0.05% (wt/vol) crystal violet and counted manually.

### 2.8. ChIP assay

ChIP assay was performed as previously described [33]. Briefly, CaCo-2 cells were treated with 1% formaldehyde for 10 min to crosslink proteins to DNA. Cell lysates were then isolated and sonicated to shear DNA into fragements between 200 and 1000 base pairs. The shared DNA was subjected to immunoprecipitation using the Chromatin Immuno-precipitation Assay Kit (EMD Millipore) with either normal IgG or a TCF4-specific antibody, following the manufacturer’s instructions. Primers CH3aF and CH3aR (Supplemental Table S1) were used for PCR amplification of the eIF3a promoter from ChIP samples.

### 2.9. Patient characteristics

A total of 22 colon cancer patients were included in the study, comprising 5 males (22.7%) and 17 females (77.3%), with a mean age of 66 years (range: 20–87). Major demographic and clinical characteristics are presented in Supplemental Table S1. Benign polyps were also collected from 8 patients, including 4 males (50%) and 4 females (50%), with a mean age of 66.8 years (range: 32–83). The major characteristics of these patients are also provided in Supplemental Table S1.

### 2.10. TCGA patient-data set analyses

Gene expression data for eIF3a and APC somatic mutation data were downloaded from the GDC TCGA (The Cancer Genome Atlas) Colon Adenocarcinoma (COAD) cohort via the UCSC Xena browser [34]. After cleaning and combining the data, a dataset of 658 colon cancer patients was obtained, including 440 with APC mutations and 218 APC wild-type. Among the 440 APC mutations, 155 were frameshift, 241 had premature stop codons, and 44 were missense mutations.

For eIF3a expression, normalized reads transformed to log2 (FPKM+1) were used, where FPKM stands for fragments per kilobase of exon per million mapped reads. Data analyses were conducted using R (version 4.3.0). The mean eIF3a expression levels of the APC wild-type and mutant groups were calculated and compared using Welch’s two-sample *t*-test, with statistical significance set at p < 0.05.

### 2.11. Xenograft and spontaneous tumor animal models

The animal studies have been approved by IACUC. Female mice were used in this study, as gender was not a key factor given the lack of significant differences in eIF3a expression in human colon tumors, and female mice are generally easier to handle with more docile behavior.

For the xenograft study, ten 7-week-old female NOD/SCID mice were randomized into two groups. Each mouse was injected subcutaneously with ~1 × 10^7^ RIE/Vec cells (control group) or RIE/eIF3a1 cells (experimental group) in 0.2 ml PBS. Tumor growth was measured with a caliper every two days, starting on day 11 post-implantation. Tumor volume was calculated using the formula: volume=(length/2) × (width^2^). All mice were euthanized on day 27 post-implantation, and the tumors were excised and weighed.

For the spontaneous model, fifteen 4-week-old female APC^min/+^ mice were randomized into three groups with 5 mice in each group. After a one-week of acclimation period, mice were injected with 40 μg scrambled or eIF3a siRNAs in in-vivo-jetPEI once every 3 days for a total of 10 treatments, as previously described [35]. The treatment groups were blinded. The mice were sacrificed 2 months after the initial treatment, and their intestines were harvested for examination of tumor existence. Sections were subjected to hematoxylin and eosin (H&E) staining as well as immunohistochemistry analysis of eIF3a expression.

### 2.12. Statistical analyses

All in-vitro experiments were performed at least three times, and all in-vivo experiments had five animals per group for statistical analysis. Statistical significance was determined using either a two-tailed Student’s t-test or one-way analysis of variance (ANOVA) with Tukey’s post hoc test, where applicable. A p-value of <0.05 was considered statistically significant. Results are presented as mean ± standard deviation.

## 3. Results

### 3.1. Expression of eIF3a, b, g, and i in human colon cancers

**Fig. 1.**
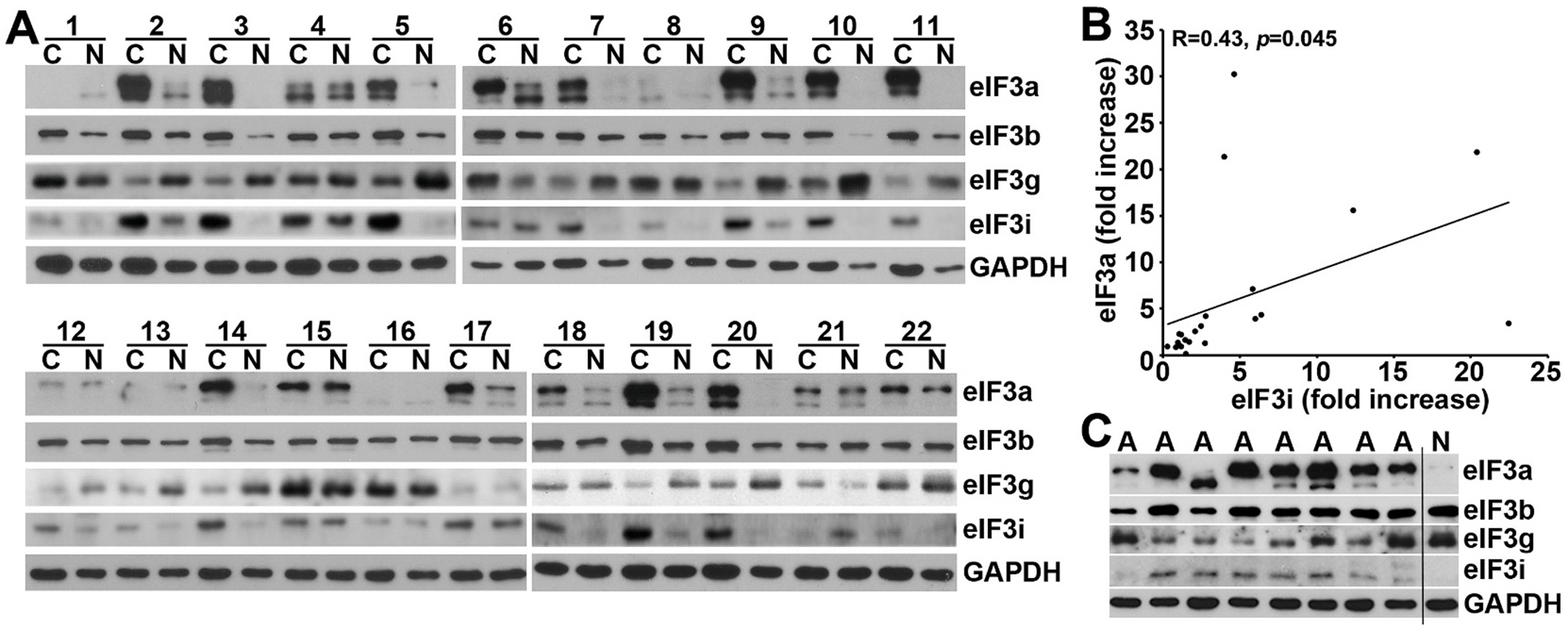
Expression of eIF3a, b, g, and i in human adenocarcinoma and adenoma colon tissues. **A.** Lysates were prepared from flash frozen paired human normal (N) and adenocarcinoma (C) tissues for Western blot analysis. GAPDH was used as a loading control. **B.** Pearson correlation coefficient analysis between eIF3a and eIF3i fold-increase in expression in cancer over normal tissues. **C.** Expression of eIF3a, b, g, and i in benign colon adenoma polyps (A) in comparison with the normal (N) tissue from patient #20 as determined using Western blot.

The eIF3 complex consists of 3 stable subcomplexes of eIF3(a:b:i:g), eIF3(c:d:e:l:k), and eIF3(f:h:m) [36] and eIF3a has been shown to serve as the docking subunit for the formation of the eIF3(a:b:i:g) subcomplex [37]. Of the eIF3(a:b:i:g) subcomplex, eIF3i has been shown to overexpress in human colon cancers [38]. Thus, we are interested in determining the expression profiles of other subunits in the eIF3(a:b:i:g) subcomplex. For this purpose, we obtained 22 matched flash frozen human normal and cancer colon tissues and performed Western blot analysis. As shown in Fig. 1A and Supplemental Fig. S1, eIF3a expression is significantly increased by ~6 folds in cancer compared with the matched normal tissues. However, no significant difference in eIF3a upregulation was observed between patients of different gender or race (Table S1). eIF3i expression is also significantly increased by ~5 fold in cancer compared with their matched normal tissues (Fig. 1A and Fig. S1A), consistent with the previous finding [38], Interestingly, the expression of both eIF3b and eIF3g did not change significantly between cancer and normal tissues.

Correlation analysis revealed a significant positive association between eIF3a and eIF3i overexpression in human colon cancers (Fig. 1B). Additionally, the expression of both eIF3a and eIF3i but not eIF3b and eIF3g is also increased in benign adenoma polyps (Fig. 1C and Fig. S2). Thus, eIF3a and eIF3i in the eIF3(a:b:i:g) subcomplex may be related to colon cancer and are up-regulated together prior to the formation of malignant colon tumors.

### 3.2. Association of eIF3a expression with intestinal epithelial cell transformation

**Fig. 2.**
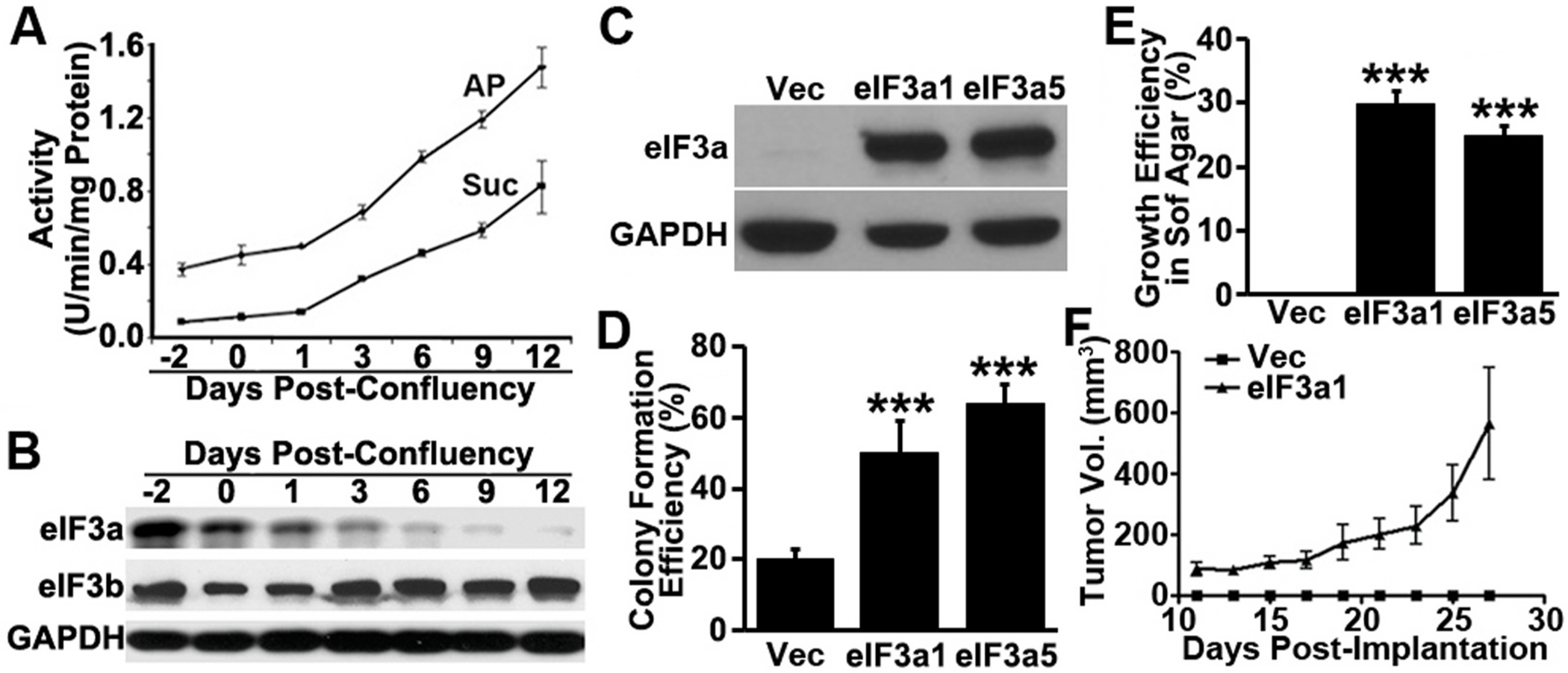
eIF3a expression in differentiated CaCo-2 cells and tumorigenic function of eIF3a. **A.** Alkaline phosphatase (AP) and sucrase (Suc) activities in CaCo-2 cells at different densities. **B**. Western blot analysis of eIF3a, eIF3b, and GAPDH control in CaCo-2 cells at different densities. **C-E.** Cell-based analysis of oncogenic function of eIF3a. Stable RIE-1 clones with eIF3a over-expression (eIF3a1 and eIF3a5) or transfected with vector control (Vec) were subjected to Western blot analysis of eIF3a and GAPDH control (C), clonogenic assay (D), and anchorage-independent growth assay in soft agar (E). **F.** In-vivo tumorigenic assay. The stable RIE clone with eIF3a over-expression (eIF3a1) or with empty vector control (Vec) were inoculated into female NOD/SCID mice followed by analyses of the growth of xenograft tumors. (***p < 0.001).

To investigate the potential oncogenic role of eIF3a in intestinal epithelial cells, we first examined its expression profile in the human colon cancer cell line CaCo-2 that can differentiate into intestine like epithelial cells and lose cancer phenotype upon confluency as indicated by expression of brush-border enzymes such as alkaline phosphatase (AP) and sucrase, differentiation markers of colon epithelial cells [27,39]. As shown in Fig. 2A–B, upon confluency as indicated by the increased expression of AP and sucrase, eIF3a expression dissipated, similar as eIF3i expression shown in the previous study [38]. However, eIF3b expression did not change, consistent with our observation using human colon cancer and normal tissues shown above.

### 3.3. eIF3a expression drives tumorigenesis

To determine the role of eIF3a in colon tumorigenesis, we used untransformed rat intestinal epithelial cells (RIE) and determined if eIF3a over-expression alone could transform these cells. RIE was chosen because it is well characterized and widely used for studying colon tumorigenesis. Two stable clones with eIF3a over-expression (RIE/eIF3a1 and RIE/eIF3a5) along with a vector-transfected control clone (RIE/Vec) were established (Fig. 2C) and tested for their proliferation and anchorage-independent growth. Fig. S3A shows that the RIE/eIF3a1 and RIE/eIF3a5 cells lost their contact-inhibition in proliferation and formed foci, compared with the RIE/Vec cells that grow in monolayer as expected. Furthermore, the RIE/eIF3a1 and RIE/eIF3a5 cells are more clonogenic than the control RIE/Vec cells (Fig. 2D and Fig. S3B) and they are able to grow in soft agar whereas the control RIE/Vec cells cannot (Fig. 2E and Fig. S3C). Thus, ectopic eIF3a over-expression effectively transformed RIE cells.

**Fig. 3.**
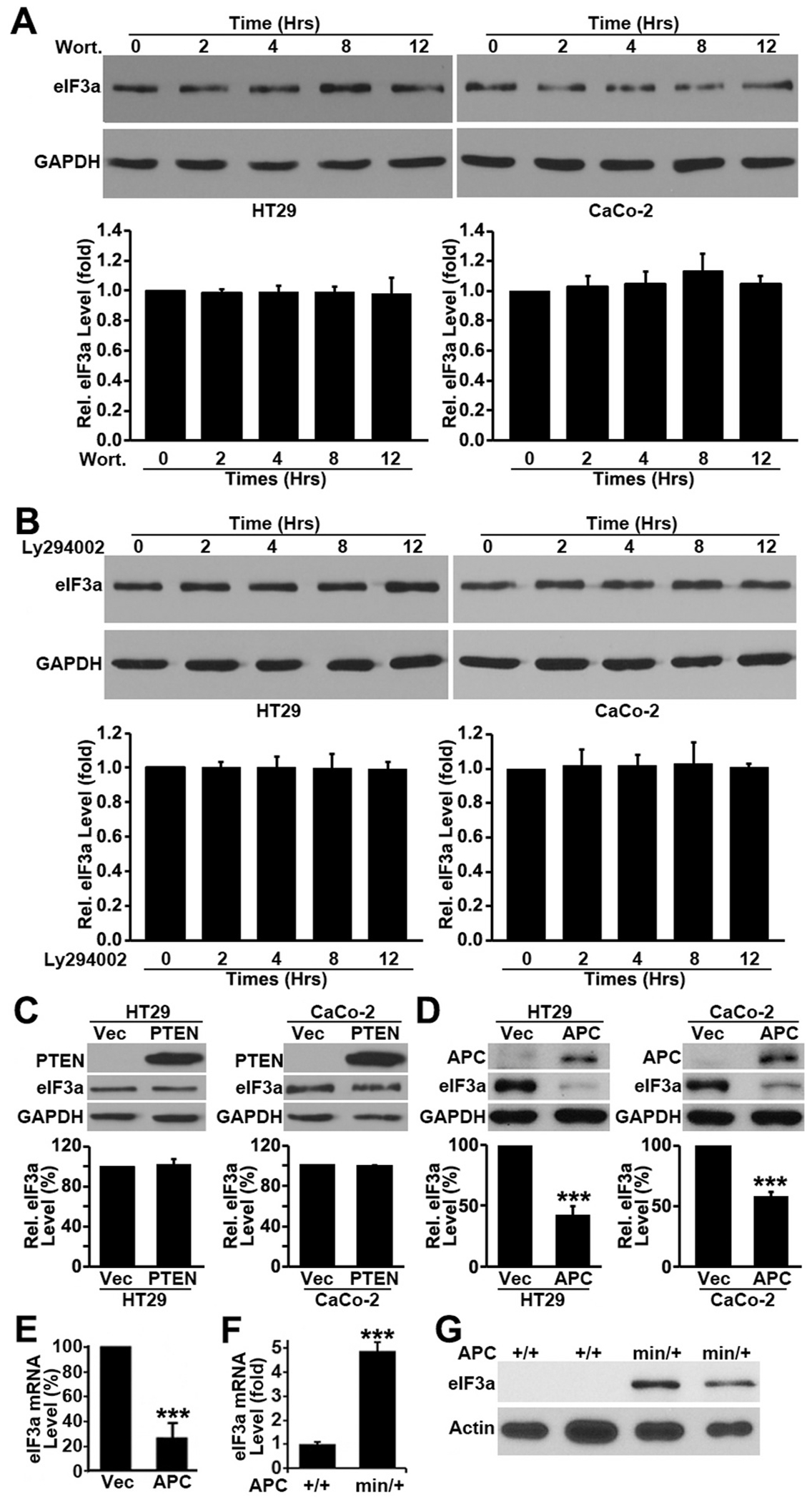
Regulation of eIF3a expression. **A-C**. Effect of PI3K inhibitors or PTEN expression on eIF3a expression. HT29 cells were treated with Wortmannin (A) or LY294002 (B) for various times, or transiently transfected with PTEN cDNA or vector control (C) followed by Western blot analysis of eIF3a, PTEN and GAPDH control. **D-E**. Effect of APC expression on eIF3a expression. HT29 and CaCo-2 cells were transiently transfected with the cDNA encoding the wild-type APC or vector control followed by Western blot analysis of eIF3a, APC and GAPDH control (D) or real-time RT-PCR analysis of eIF3a mRNA in CaCo-2 cells (E). **F-G**. APC status and eIF3a expression *in vivo*. Total RNAs or lysates were prepared from whole intestinal samples of APC^min/+^ and APC^+/+^ mice and subjected to quantitative RT-PCR (F) and Western blot (G) analysis of eIF3a. The eIF3a mRNA level in APC^min/+^ were normalized to that in the wild type control APC^+/+^ mice and Western blot analysis was performed using samples from 2 individual APC^min/+^ and 2 control APC^+/+^ mice. (***p < 0.001).

We next examined tumorigenic potential using the RIE/eIF3a1 cell as a representative model compared with the RIE/Vec control cell in immune deficient NOD/SCID mice. For this purpose, equal numbers of RIE/eIF3a1 and RIE/Vec cells were injected subcutaneously into two randomized groups of mice followed by observations. As shown in Fig. 3F, tumors were formed from RIE/eIF3a1 cells and palpable on day 11 after implantation and they grow exponentially. However, no tumors were formed in the mice implanted with the RIE/Vec control cells. The average size of the tumors from RIE/eIF3a1 cells reached ~550 mm^3^ at ~4 weeks post-implantation and the average weight of the dissected RIE/eIF3a1 tumors (Fig. S3D) are 0.66 ± 0.45 g at the end of the study. No dissectible tumors could be collected from the control group. Hence, we conclude that eIF3a over-expression drives tumorigenesis of RIE cells.

### 3.4. Regulation of eIF3a expression by APC

The above findings are very intriguing, and it is of interest to investigate how eIF3a expression is increased in colon cancers and what regulates its expression that drives tumorigenesis. Following extensive investigation on several pathways that are involved in tumorigenesis such as PI3K and MAPK pathways, we found that eIF3a expression was regulated by APC, not by others. For example, eIF3a expression was not altered by treatment with PI3K inhibitor, Wortmannin or LY294002 (Fig. 3A–B) nor by PTEN over-expression (Fig. 3C). However, over-expressing the wild-type APC gene drastically reduced eIF3a expression at both protein (Fig. 3D) and mRNA (Fig. 3E) levels in human colon cancer HT29 and CaCo-2 cells, which are known to have mutated APC gene. APC, a tumor suppressor gene that is mutated in >80% sporadic colon adenoma and cancers [40], regulates the expression of a set of genes that control cell growth and tumorigenesis. These findings suggest that APC may be a major upstream regulator of eIF3a and that APC mutations may up-regulate eIF3a which, in turn, contributes to colon tumorigenesis.

To validate above findings and to determine if APC possibly regulates eIF3a expression *in vivo*, we took advantage of the APC^min/+^ mice, which have APC mutation and develop multiple adenomas [41], and tested eIF3a expression in the intestines of these animals in comparison with wild-type APC^+/+^ mice using quantitative RT-PCR and Western blot analyses. As shown in Fig. 3F, the intestine of APC^min/+^ mice have ~5 fold more eIF3a mRNA than the APC^+/+^ mice. The eIF3a protein in APC^+/+^ mice is not detectable while it is drastically up-regulated in the APC^min/+^ (Fig. 3G). The finding of undetectable eIF3a protein in intestines of APC^+/+^ mice is consistent with our previous observation that eIF3a is not detectable by Western blot in whole intestines of adult wild-type mice [27]. Thus, the wild-type APC may suppress eIF3a expression in intestines and its mutation or Wnt activation may release the suppression, causing the eIF3a level to rise.

### 3.5. Regulation of eIF3a expression by Wnt/β-catenin pathway

**Fig. 4.**
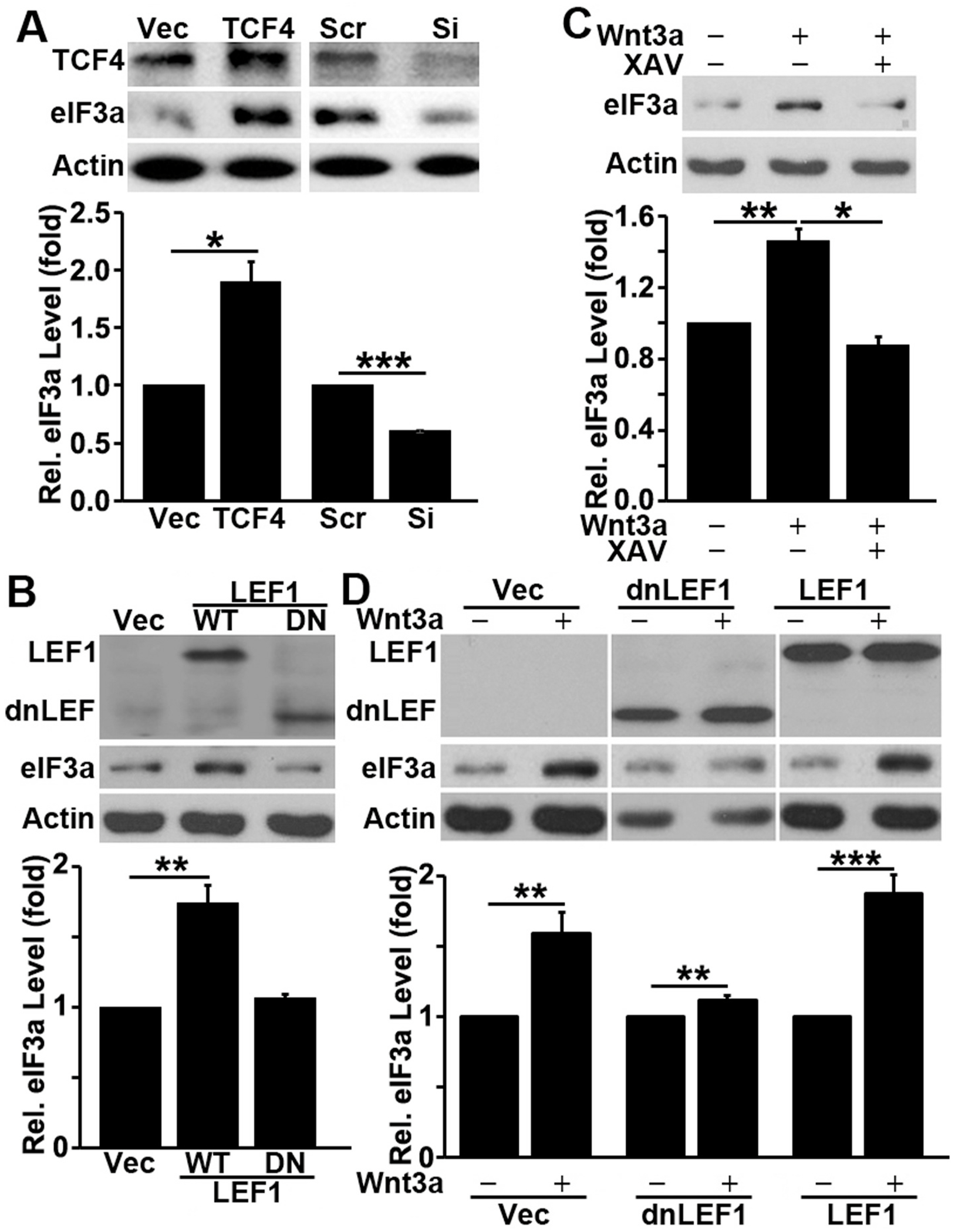
Regulation of eIF3a expression by Wnt signaling. **A-B**. Effect of TCF4 or LEF1 on eIF3a expression. CaCo-2 cells were transiently transfected with TCF4 cDNA or siRNA along with their respective vector and scrambled siRNA controls (A) or with wild-type (WT) or dominant negative mutant (DN) LEF1 cDNA along with its vector control (B) followed by Western blot analysis of eIF3a, TCF4, LEF1, or actin control. **C-D**. Effect of Wnt signaling pathway activation on eIF3a expression. HEK293 cells were serum-starved for 36 h followed by treatment with Wnt3a in the absence or presence of tankyrase inhibitor XAV939 (C) or transiently transfected first by vector, wild-type or dominant negative (dn) LEF1 before serum starvation and Wnt3a treatment (D), followed by Western blot analysis of LEF1, eIF3a or actin control.

To further understand the possible regulation of eIF3a expression by APC in the Wnt/β-catenin signaling pathway, we first tested the effect of TCF4 on eIF3a expression by over-expressing or knocking down TCF4 in CaCo-2 cells. As shown in Fig. 4A, TCF4 over-expression and knockdown significantly increased and decreased eIF3a expression, respectively. Over-expressing the wild-type but not the mutant LEF1 also increased eIF3a expression (Fig. 4B).

Next, we tested if activating Wnt/β-catenin pathway stimulates eIF3a expression. For this purpose, we took advantage of HEK293 cells, which are very responsive to exogenous Wnt and widely used as a tool for studying Wnt/β-catenin signaling. Briefly, HEK293 cells cultured in serum-free media were treated with Wnt3a in the absence or presence of tankyrase inhibitor XAV939, which causes β-catenin degradation and inhibits β-catenin-mediated transcription [42], followed by Western blot analysis of eIF3a. As shown in Fig. 4C, Wnt3a treatment stimulated eIF3a expression, which was intercepted by XAV939. Furthermore, expressing the dominant negative but not the wild-type LEF1 also successfully inhibited the Wnt3a-stimulated eIF3a expression in HEK293 cells (Fig. 4D). Together, we conclude that APC and Wnt/β-catenin signaling likely regulate eIF3a expression.

### 3.6. Regulation of eIF3a promoter activity by Wnt/β-catenin pathway

**Fig. 5.**
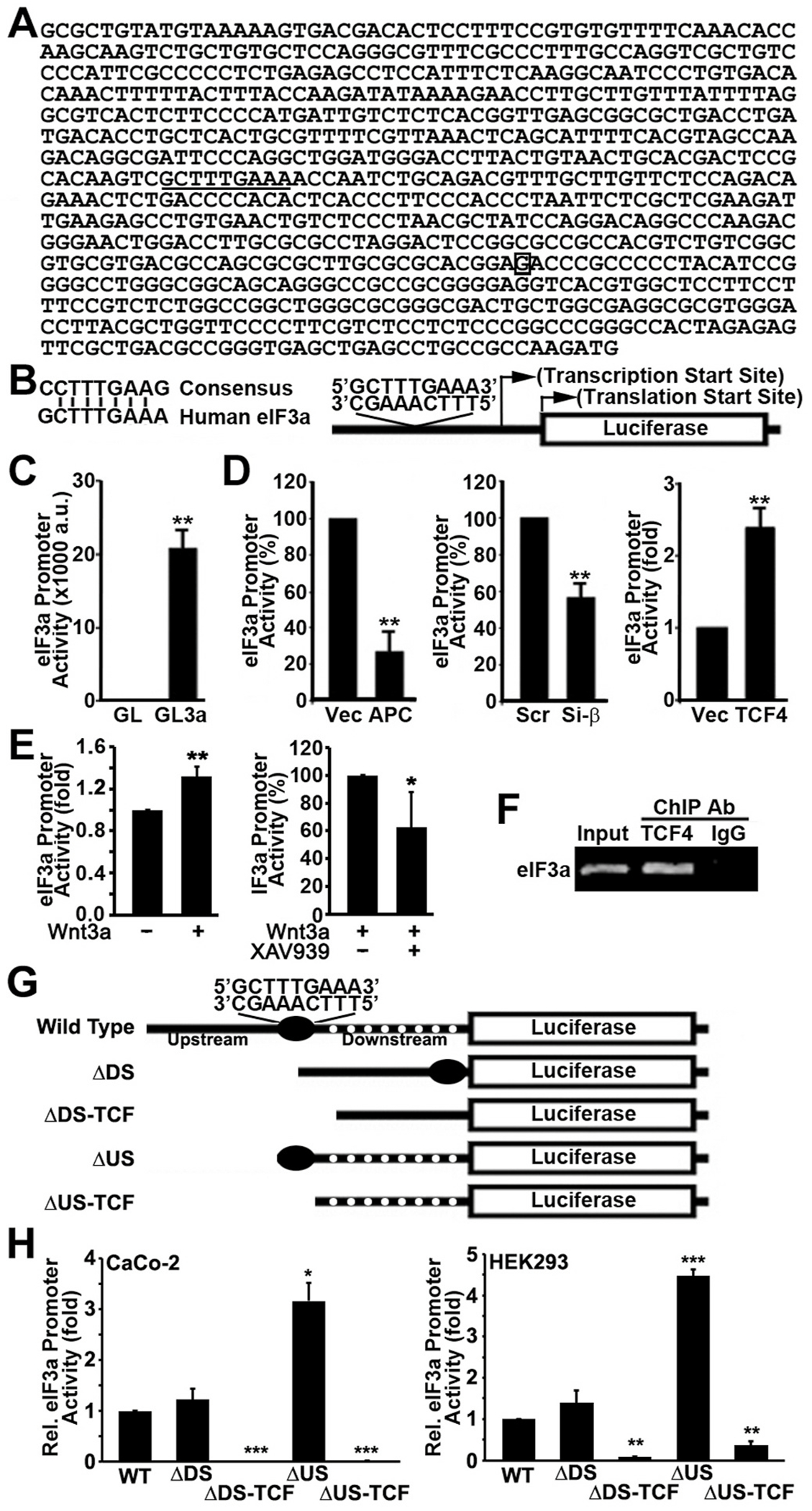
Sequence and activity of eIF3a promoter. **A**. Promoter sequence of human eIF3a. The putative LEF/TCF binding site GCTTTGAAA (underlined) is 279 bases upstream of the transcription start site (boxed G) and 426 bases upstream of the translation initiation codon ATG. **B**. Comparison between the consensus and the putative LEF/TCF binding sequences in human eIF3a gene and the schematic diagram of the luciferase reporter construct. **C-D**. Basal eIF3a promoter activity and its regulation by APC, β-catenin, and TCF4. Promoter construct (GL3a) shown in panel B along with a promoter-less control construct (GL) were transiently transfected into CaCo-2 cells (C) or CaCo-2 cells with over-expression of APC, TCF4 or with β-catenin knockdown (D) for determination of luciferase activity. Vector-transfected controls for APC and TCF4 overexpression or scrambled siRNA control for β-catenin knockdown were also tested, β-galactosidase was used to control transfection efficiency. a.u. = arbitrary units. **E.** Effect of Wnt3a and tankrase inhibitor on the promoter activity of eIF3a in HEK293 cells. **F.** ChIP assay of eIF3a promoter using TCF4 antibody or control normal IgG. **G.** Schematic drawing of deletion constructs of eIF3a promoter sequence. **H.** Relative promoter activity of wild-type and deletion mutant eIF3a promoters in CaCo-2 and HEK293 cells. (***p < 0.001, **p < 0.01, *p < 0.05).

Sequence analysis of the human eIF3a TATA-less promoter [43,44] revealed a potential LEF/TCF-binding site, located 219 bases upstream of the transcription start site (Fig. 5A). Although the first and the last bases in the potential LEF/TCF binding site in eIF3a promoter vary from those in the consensus sequence (C and G vs G and A, respectively, Fig. 5B), these differences have been shown previously not to alter the binding affinity to the LEF/TCF transcription factors [45,46].

To determine the role of the LEF/TCF/β-catenin transcription factors in regulating eIF3a expression, we engineered a reporter construct (pGL3a) by cloning eIF3a promoter into the pGL3 luciferase reporter vector (Fig. 5B) and transiently transfected it into CaCo-2 cells to determine eIF3a promoter activity. Fig. 5C shows that eIF3a promoter has a strong basal activity in CaCo-2 cells, consistent with activated Wnt/β-catenin signaling in this cell line. Next, we altered the expression of APC, TCF4, and β-catenin in CaCo-2 cells, transiently transfected the eIF3a promoter construct pGL3a into these cells and determined the change in eIF3a promoter activity. As shown in Fig. 5D, expressing the wild-type APC or knocking down endogenous β-catenin significantly suppressed eIF3a promoter activity. Consistently, TCF4 over-expression significantly increased eIF3a promoter activity. These studies were also performed using additional cell lines and similar results were observed (Fig. S4). Thus, the eIF3a promoter is likely under Wnt/β-catenin control and the regulation of eIF3a expression by APC, TCF4, and β-catenin is cell line-independent.

Next, we tested if activating Wnt/β-catenin pathway stimulates eIF3a promoter activity in HEK293 cells as described above. As shown in Fig. 5E, Wnt3a treatment stimulated eIF3a promoter activity and the tankyrase inhibitor XAV939 reduced Wint3a-stimulated eIF3a promoter activity.

Finally, we performed ChIP assay to determine if the transcription factor TCF4 may bind to the endogenous eIF3a promoter in CaCo-2 cells. Fig. 5F shows that the promoter sequence of eIF3a was successfully immunoprecipitated by TCF4 antibody but not by an irrelevant control IgG. Thus, the Wnt/β-catenin-stimulated eIF3a expression is likely due to direct binding of β-catenin/TCF to the putative LEF/TCF-binding site in the promoter sequence of eIF3a gene.

### 3.7. Mapping the promoter sequence of eIF3a

To demonstrate that the putative LEF/TCF binding site (CTTTGAAA) is functional in activating eIF3a expression and to evaluate potential existence of other regulatory elements, we performed deletion mapping analysis of the eIF3a promoter. For this purpose, four deletion constructs were engineered (F. 5G) and were transfected into CaCo-2 cells along with the wild-type construct for determination of promoter activity. As shown in Fig. 5H, deletion of the sequence downstream of the putative LEF/TCF-binding site had no effect on the promoter activity. However, further deletion to remove putative LEF/TCF-binding sequence CTTTGAAA abolished the promoter activity. Interestingly, deletion of the upstream sequence increased the promoter activity, suggesting that there may be an inhibitory element upstream of the putative LEF-TEF-binding site. Further deletion to remove the putative LEF/TCF-binding site completely abolished the promoter activity of eIF3a. Similar promoter activity profiles of these constructs were also observed when HEK293 cells were used (Fig. 5H). These findings suggest that the putative LEF/TCF-binding sequence CTTTGAAA in eIF3a promoter is likely functional and it may represent an important element responsible for eIF3a expression in colon cancer cells.

### 3.8. Association between β-catenin and eIF3a expression in human colon cancer tissues

**Fig. 6.**
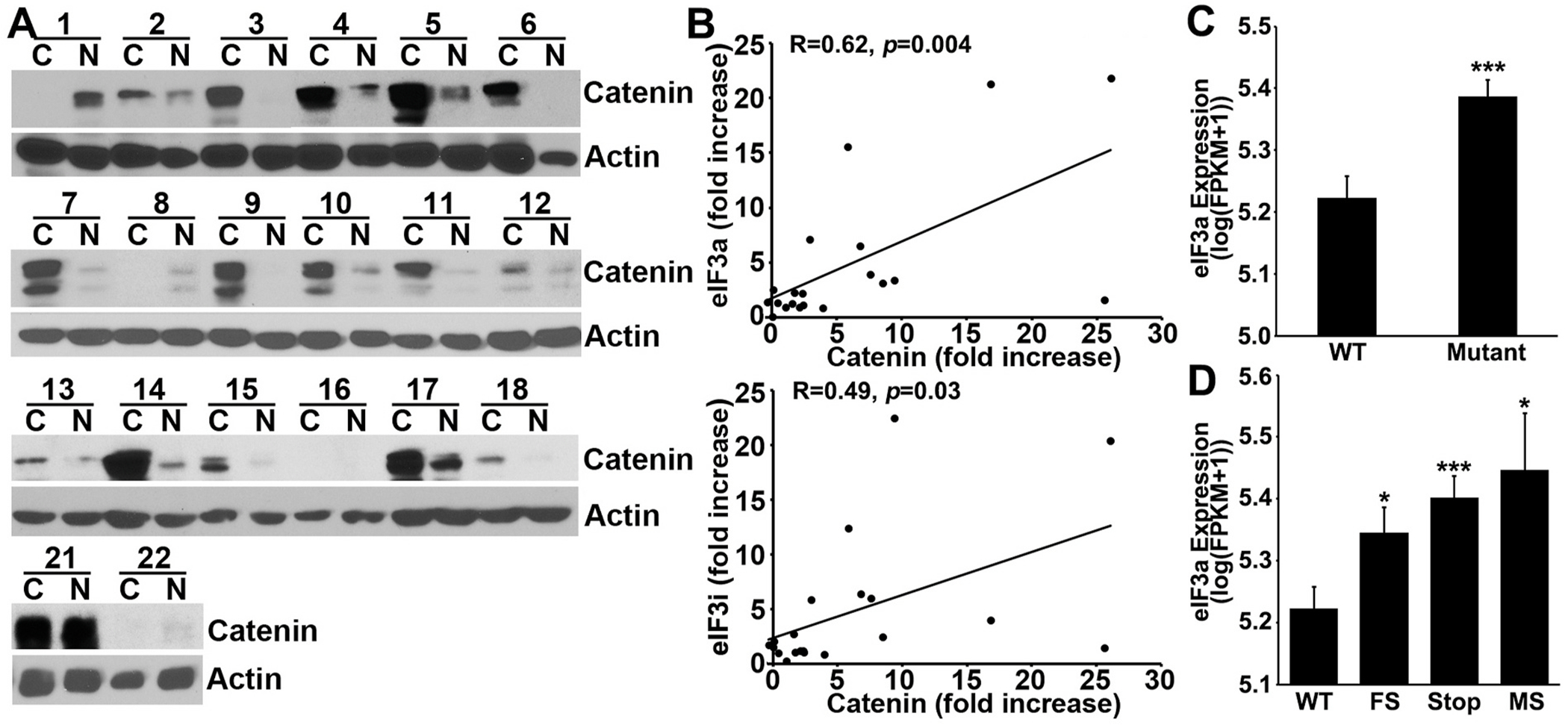
eIF3a association with β-catenin expression in human colon adenocarcinoma tissues and with APC mutations. **A.** Western blot analysis of β-catenin and actin control in the 20 matched pairs of human normal (N) and adenocarcinoma (C) tissues. **B**. Pearson correlation analysis of the fold-increase in expression in cancer over normal tissues between eIF3a and β-catenin and between eIF3i and β-catenin. **C-D**. Association between APC mutation and eIF3a expression in the human colon cancer TCGA dataset. WT, wild-type; FS, frameshift mutation; Stop, gaining premature stop codon; MS, missense mutation.

Next, we determined the expression of β-catenin in the matched pairs of human colon cancer and normal tissues using Western blot analysis. Only 20 pairs of the original 22 pairs of tissues were available for this study. As shown in Fig. 6A and Fig. S5, β-catenin expression is upregulated by ~6 fold in the colon cancer tissues compared with their matched normal tissues. We next analyzed the association between eIF3a and β-catenin upregulation in these samples as we described above for eIF3a and eIF3i (see Fig. 1). As shown in Fig. 6B, the increase in eIF3a expression positively associates with that of β-catenin. Similarly, the increased expression of eIF3i also positively associates with that of β-catenin. However, it is noteworthy that the quantification of β-catenin was performed in different experiments from that of eIF3a and eIF3i. While the association analyses using these datasets from different experiments have limitations, the fact that they were from the same samples and showed strong correlation suggests that Wnt/β-catenin signaling may contribute to the upregulation of both eIF3a and eIF3i in human colon cancers.

### 3.9. Association between APC mutation and eIF3a expression

Based on above findings, we hypothesized that APC mutations in human colon cancer tissues would upregulate the expression of eIF3a. To test this hypothesis, we analyzed the publicly available gene expression RNAseq FPKM (Fragment per Kilobase of exon per Million) and somatic mutation datasets (see Materials and Methods). As shown in Fig. 6C, the eIF3a expression level is significantly increased in samples with APC mutation compared with that of wild-type APC. Detailed analyses also showed that the increased eIF3a expression is irrespective of the type (frameshift, premature stop codon, or missense) of APC mutation (Fig. 6D). These findings suggest that the Wnt/β-catenin regulation of eIF3a expression likely occurs clinically.

### 3.10. eIF3a knockdown inhibits spontaneous tumorigenesis in APC^mm/+^ mice

**Fig. 7.**
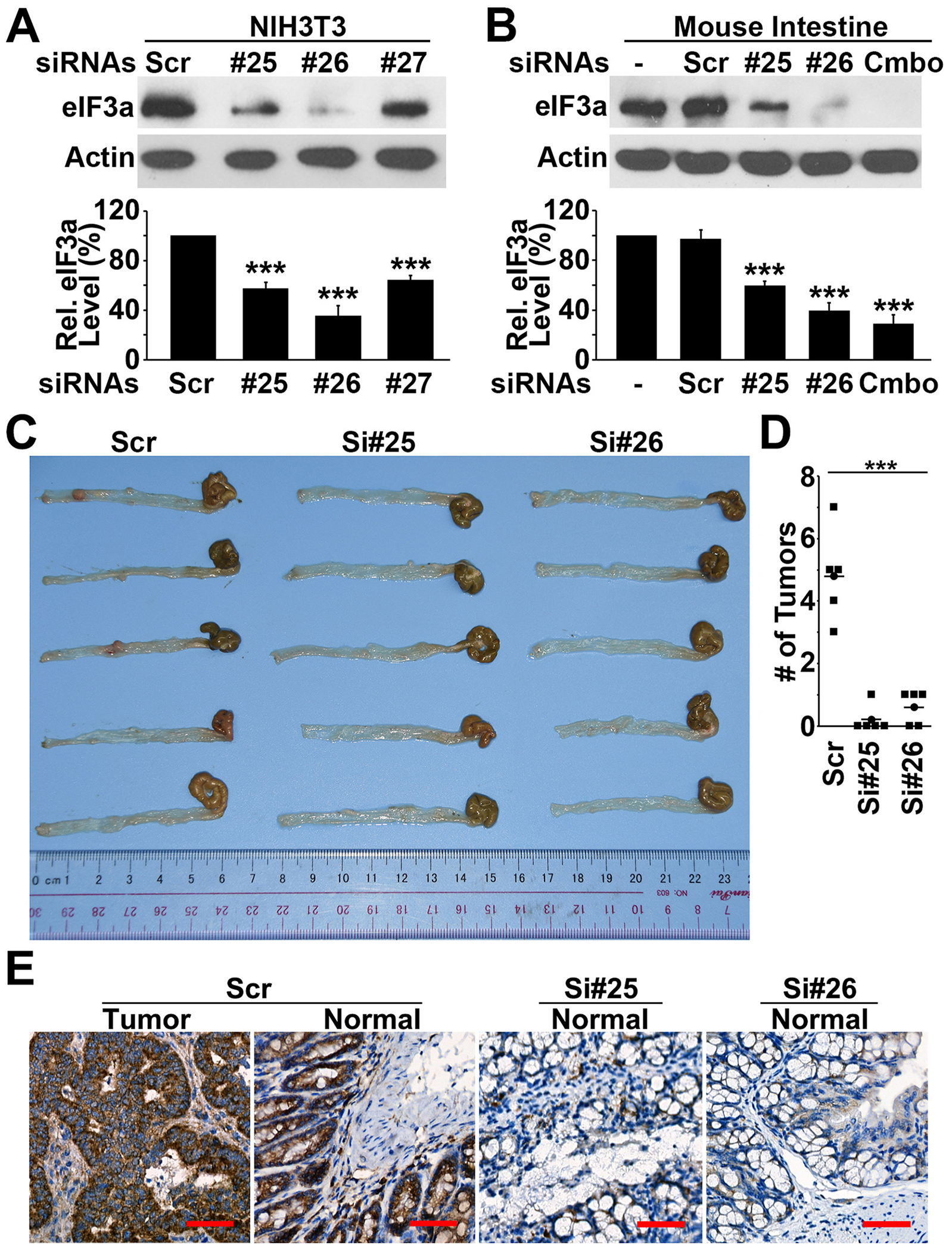
eIF3a knockdown inhibits APC mutation-induced colon tumor production. **A-B.** Activity of three different siRNAs in knocking down eIF3a expression in NIH3T3 cells (A) and in mice (B). **C-D.** Effect of eIF3a knockdown on tumor formation in APCmin/+ mice. (***p < 0.001). **C**. IHC staining of eIF3a in intestinal tissues of APC^min/+^ mice treated with scrambled control or eIF3a siRNAs. Scale bar, 50 μm.

As shown above, eIF3a overexpression induces tumorigenesis and APC mutation up-regulates eIF3a expression. Thus, it is possible that eIF3a may mediate APC mutation-induced tumorigenesis. To test this hypothesis, we determined if intercepting APC mutation-induced eIF3a expression could suppress APC mutation-induced tumorigenesis using eIF3a knockdown. For this purpose, we first tested three siRNAs using NIH3T3 cells and found two siRNAs (#25 and #26) that can effectively knock down mouse eIF3a expression (Fig. 7A). Both siRNAs could also effectively inhibit eIF3a expression in mouse intestine when injected intravenously into mice (Fig. 7B). Next, these siRNAs were injected intravenously into APC^mm/+^ mice followed by examination of spontaneous tumor formation in intestines. As shown in Fig. 7C–D, both siRNAs essentially eliminated tumor production compared with the control siRNA-treated mice. Fig. 7E shows the colon tissue sections in the control and the siRNA-treated mice. IHC staining of these sections showed significant reduction of eIF3a expression in the intestines of mice injected with eIF3a siRNAs, indicating that the reduced tumor production was likely due to eIF3a knockdown. Interestingly, the body weight of these mice did not change significantly following eIF3a knockdown (data not shown), suggesting that eIF3a siRNA did not cause significant adverse effect to these mice. Thus, we conclude that eIF3a plays an important role in intestinal/colon tumorigenesis and inhibiting eIF3a may intercept APC mutation-induced colon tumor production.

## 4. Discussion

In this study, we not only showed that eIF3a expression is up-regulated in human colon cancer tissues and contributes to colon tumorigenesis, we also demonstrated that its expression is regulated by the Wnt/β-catenin signaling pathway and that inhibiting eIF3a expression can intercept APC mutation-induced tumorigenesis in intestinal tract. These findings suggest that eIF3a may be an oncogenic mediator in APC mutation-induced colon cancers and can be used as a target for therapeutic intervention of colon cancers.

Although we have clearly shown that eIF3a is regulated by Wnt/β-catenin signaling pathway in cell-based studies and that APC mutation up-regulates eIF3a expression in the APC^mm/+^ animal model, it remains to be determined if the increased eIF3a expression in human colon cancers is due to APC mutation in these cancers. Since APC mutation occurs in >80% sporadic colorectal adenomas and adenocarcinomas [40], it is possible that most of the cancer tissues we studied have APC mutations. The finding that 18 of the 22 cases (82%) in this study had increases in eIF3a expression is consistent with the rate of APC mutation. Indeed, analyses of publicly available datasets confirmed that APC mutations associates with eIF3a expression. Thus, it is likely that APC mutation causes eIF3a upregulation in human colon cancer.

As subunits in the eIF3(a:b:i:g) subcomplex, only eIF3a and eIF3i, but not eIF3b and eIF3g, are overexpressed in human colon cancers compared with their matching normal tissues. While the increased eIF3a expression may be due to APC mutation and/or Wnt activation, it is unclear whether eIF3i may also be activated by APC mutation and/or Wnt activation. Analysis of the eIF3i promoter sequence, indeed, revealed a potential TCF/LEF-binding site (ACTCCAAAGA) 54 bases upstream the transcription start site. Thus, it is possible that both eIF3a and eIF3i are under Wnt/β-catenin signaling regulation. The finding that the increased expression of eIF3a and eIF3i strongly associates with each other in 19 of the 22 matched pairs of human tissues is consistent with the conclusion that both eIF3a and eIF3i may be regulated similarly in colon cancers. Interestingly, we have shown previously that eIF3i also regulates β-catenin activation by increasing COX-2 synthesis and production of PGE2 [38]. Thus, it is also possible that eIF3i participates in the feedforward loop of the Wnt/β-catenin signaling pathway.

The finding that the enforced eIF3a expression transformed the normal intestinal epithelial RIE cells into tumorigenic ones is consistent with previous observations that eIF3a knockdown reverses the malignant phenotype of human cancer cells [17] and eIF3a over-expression transforms mouse fibroblasts [25]. These findings, together with the observation that eIF3a expression is up-regulated in the pre-malignant adenoma tissues, suggest that eIF3a may be a proto-oncogene. Although the molecular mechanism of eIF3a action in oncogenesis remains elusive, it is tempting to speculate that eIF3a may suppress synthesis of tumor suppressor proteins at the level of mRNA translation. Consistent with this speculation, it has been shown previously that eIF3a suppresses synthesis of several DNA damage repair proteins [19–22] and of a tumor suppressor p27 [17]. eIF3a inhibition in synthesis of DNA repair proteins may sensitize and help predispose epithelial cells to carcinogen-induced tumorigenesis [47].

The observation that eIF3a knockdown using siRNA significantly reduces intestinal tumor formation in APC^mm^ mice supports the above conclusion that eIF3a is a proto-oncogene. It may be a major contributor to APC mutation-induced colon tumorigenesis. This finding also suggests that eIF3a can be established as a potential target for treating colon cancers and to intercept APC mutation-induced tumorigenesis. Systematically knocking down eIF3a expression using siRNAs did not cause apparent adverse effect to the mice, further confirms eIF3a as a potential target for drug discovery. Future studies are necessary to characterize eIF3a as a drug target for colon cancers.

In addition to eIF4E and now eIF3a, which have been shown to be tumorigenic, other eIFs such as eIF4G have also been shown to be able to transform NIH3T3 fibroblasts [48]. Recently, eIF5A has also been indicated in colorectal cancer by regulating Myc synthesis [49]. Interestingly, Myc translationally regulates the expression of other genes via an eIF3d-dependent initiation mechanism [50]. Thus, it is now clear that dysregulation of translational control is likely a major factor contributing to malignant transformation. Although the detailed mechanism of translational control in malignant transformation remains to be determined, the prevailing concept is that increased eIFs such as eIF4E increases the translation of mRNAs that are normally inefficiently translated and these mRNAs may encode proteins promoting cell growth [51]. However, our previous findings on eIF3a suggest that there may be an alternate mechanism where eIFs suppress translation of mRNAs encoding tumor suppressor and growth retardation proteins [17,19,20,28]. A recent study using PAR-CLIP profiling of mRNAs bound to eIF3a, b, d, and g also supports this concept [23]. The finding of eIF3a functioning as a m6A reader [52,53] may render such functions in translational regulation.

**Fig. 8.**
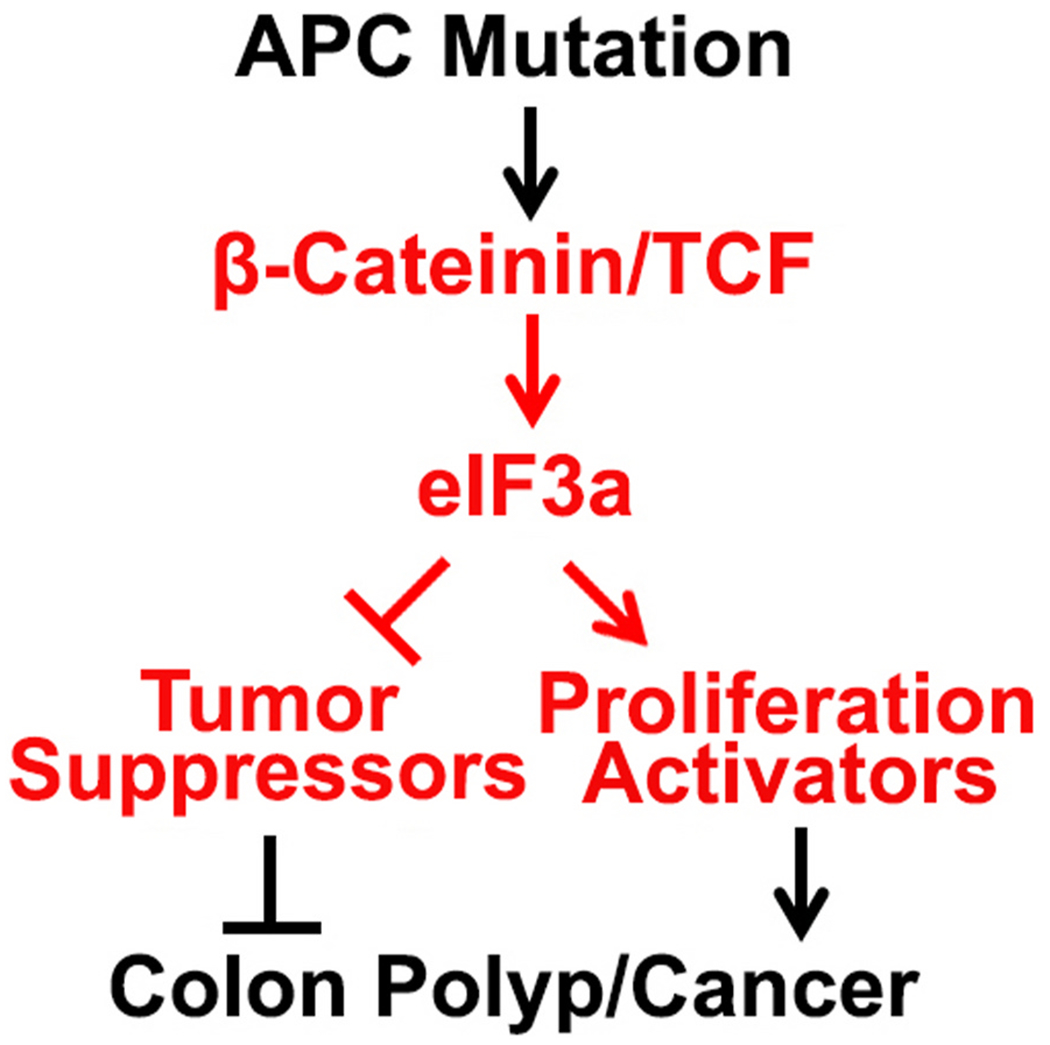
Schematic model of the speculated translational control axis in the Wnt/β-catenin signaling and colon tumorigenesis.

The best understood and extensively studied signal transduction pathway involving regulation of eIFs and translational control is the mTOR pathway [54,55]. The finding of the current study that Wnt/β-catenin signaling pathway involves in regulating eIF3a expression and that eIF3a mediates APC mutation-induced colon tumorigenesis is novel and interesting. Considering that eIF3a regulates translation of a group of mRNAs [19–23] including the one encoding the tumor suppressor p27 [17], it is tempting to speculate that eIF3a may provide a translational control axis in the Wnt/β-catenin signaling pathway, which controls gene expression at the level of protein synthesis (Fig. 8).

## Supplementary Material

Supplemental Information

## Data Availability

The data analyzed in this study were obtained from GDC TCGA (The Cancer Genome Atlas) Colon cancer (COAD) cohort via the UCSC https://xenabrowser.net/datapages/.
