# Supplementary material for: The eIF3a translational control axis in the Wnt/β-catenin signaling pathway and colon tumorigenesis
Source: Cancer Lett. Author manuscript; Available in PMC 2024 Dec 15. (PMC11646415; doi:10.1016/j.canlet.2024.217303)
Supplement: Supplemental Information [file NIHMS2038805-supplement-Supplemental_Information.pdf]

The eIF3a translational control axis in the Wnt/β-catenin signaling pathway and colon tumorigenesis

Zizheng Dong, Anuj Ojha, Lincoln Barlow, Liyun Luo, Jing-Yuan Liu, Jian-Ting Zhang*

Department of Cell and Cancer Biology, The University of Toledo College of Medicine and Life Sciences, Toledo, Ohio, USA.

Running Title: Translational control in Wnt/β-catenin signaling and colon tumorigenesis

* To whom all correspondence should be addressed: Department of Cell and Cancer Biology, The University of Toledo College of Medicine and Life Sciences, 3000 Arlington Ave, Toledo, Ohio, USA. Tel: 419-383-4161; Email: jianting.zhang@utoledo.edu

Supplemental Table S1: Primers for eIF3a promoter cloning, PCR, and CHIP Assay

Primer Name	Sequence
**eIF3aProF**	5’-GC AAG CTT CCT TCT GAG CTC ACC TTT ATT TCG TAC-3’
**eIF3aProR**	5’-CT CCA TGG CTT GGC GGC AGG CTC AGC TCA CCC-3’
**eIF3aPromut1**	5’-CT CCA TGG GAC TTG TGC GGA GTC GTG CAG-3’
**eIF3aPromut2**	5’-GCA AGC TTC AAT CTG CAG ACG TTT GCT TGT-3’
**CH3aF**	5’-GC AAG CTT CTC AGC ATT TTC ACG TAG CCA AGA-3’
**CH3aR**	5’-CT CCA TGG TCC TGG ATA GCG TTA GGG AGA CAG-3’
**eIF3aRTF**	5′-AGATGAGGACAGAGGACCTAGAC-3′
**eIF3aRTR**	5′-TCAGCATTCCGCCAGGATGA-3′
**GAPDHF**	5′-AAGGACTCATGACCACAGTCCAT-3′
**GAPDHR**	5′-CCATCACGCCACAGTTTTC-3′

Table S2. Colon Cancer and Benign Adenoma Patient Characteristics and eIF3a Expression

Characteristics	Cancer Patients	Mean eIF3a Increase (fold)	*P* value	Normal Patients	Mean eIF3a Increase (fold)	*P* value
**Total Patients**	22 (100%)	6.0±8.1		8 (100%)	3.2±0.8	
**Age (years)**	66.0±15.2			66.8±21.4		
**Gender**						
Male	5 (22.7%)	3.5±2.1	0.22	4 (50%)	3.1±0.6	0.76
Female	17 (77.3%)	6.7±9.1		4 (50%)	3.3±1.0	
**Race**						
Black	4 (18.2%)	7.8±8.1	0.68			
White	18 (81.8%)	5.6±8.1		8 (100%)		
**Histology**						
Adenocarcinoma	22 (100%)					
Adenoma				8 (100%)		

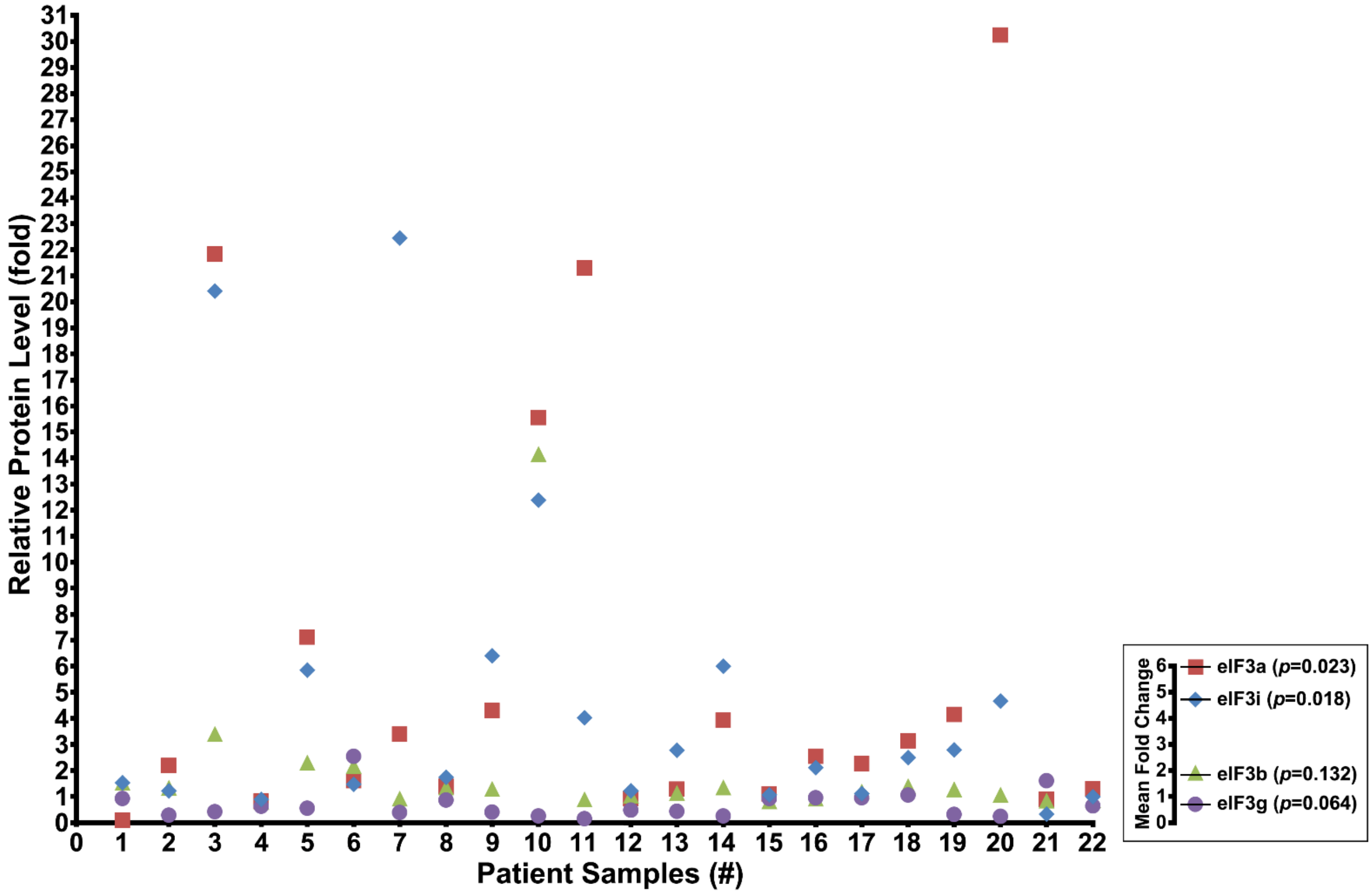

Figure S1. Fold change of eIF3a, b, g, and i in cancer relative to paired normal tissues. Each color-coded symbol represents fold change in the expression of eIF3a (■), eIF3b (▲), eIF3g (●), and eIF3i (◆) in each paired sample (cancer vs normal) as determined from Western blot in Figure 1A. The mean change of each subunit is shown in the box on the right.

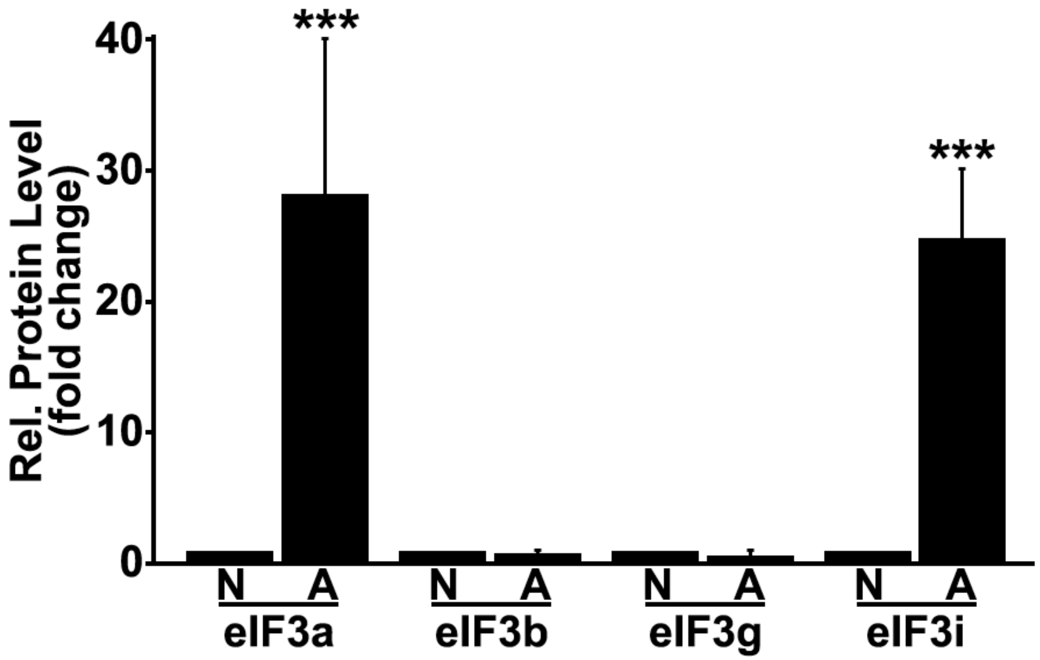

Figure S2. Fold change of eIF3a, b, g, and i in adenoma polyps. The relative protein level is the mean fold change in all eight adenoma (A) tissues normalized to that of the normal (N) tissue of patient #20. (***p<0.001).

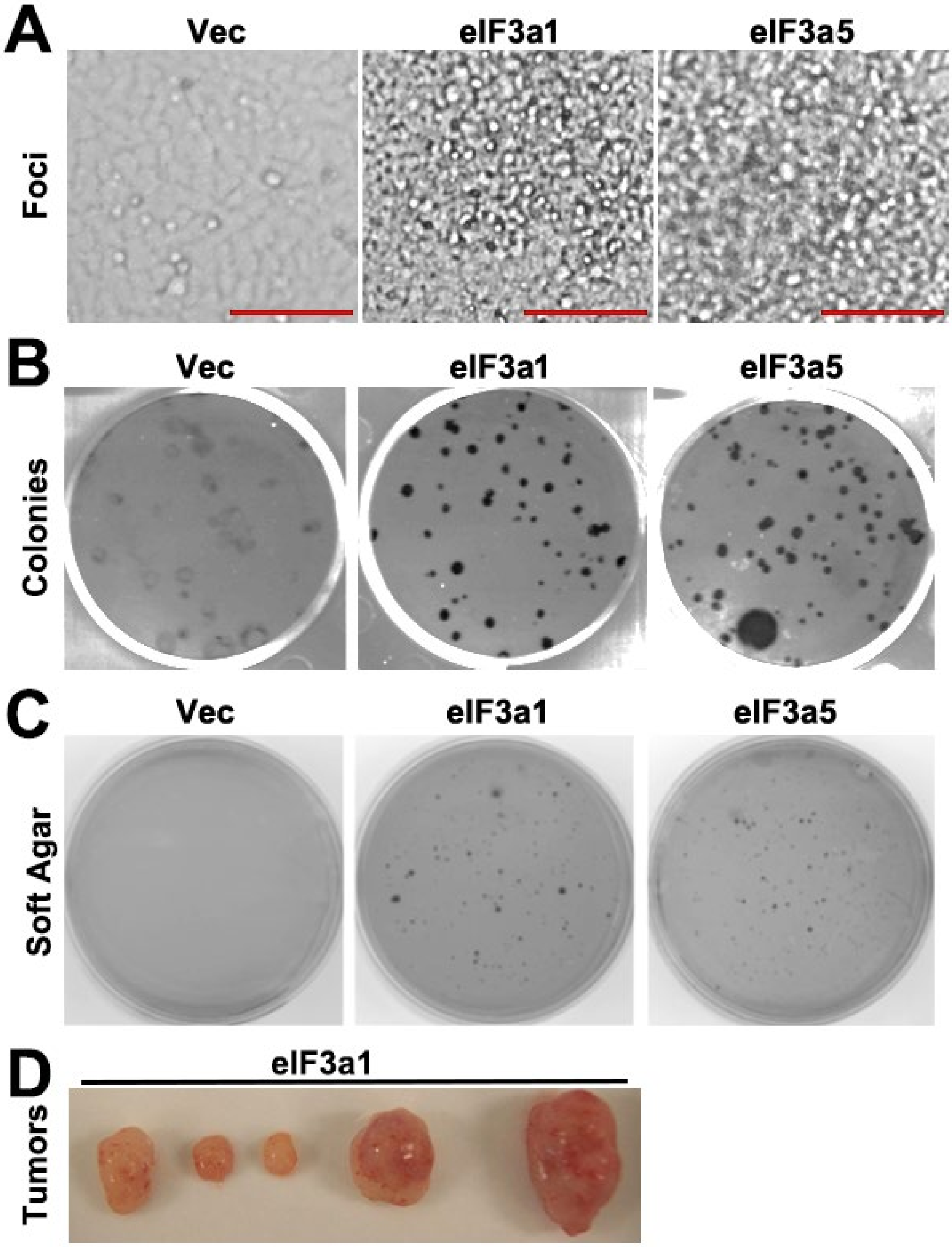

Figure S3. Oncogenic function of eIF3a. Stable RIE-1 clones with eIF3a over-expression, RIE/eIF3a1 (eIF3a1) and RIE/eIF3a5 (eIF3a5) or transfected with vector control RIE/Vec (Vec) were subjected to foci formation assay (A), clonogenic assay (B), and anchorage-independent growth assay in soft agar (C). Scale bar=25 µm. Panel D shows the xenograft tumors derived from the stable RIE/eIF3a1 clone (eIF3a1) overexpressing eIF3a. No xenograft tumor was formed from RIE/Vec control cells.

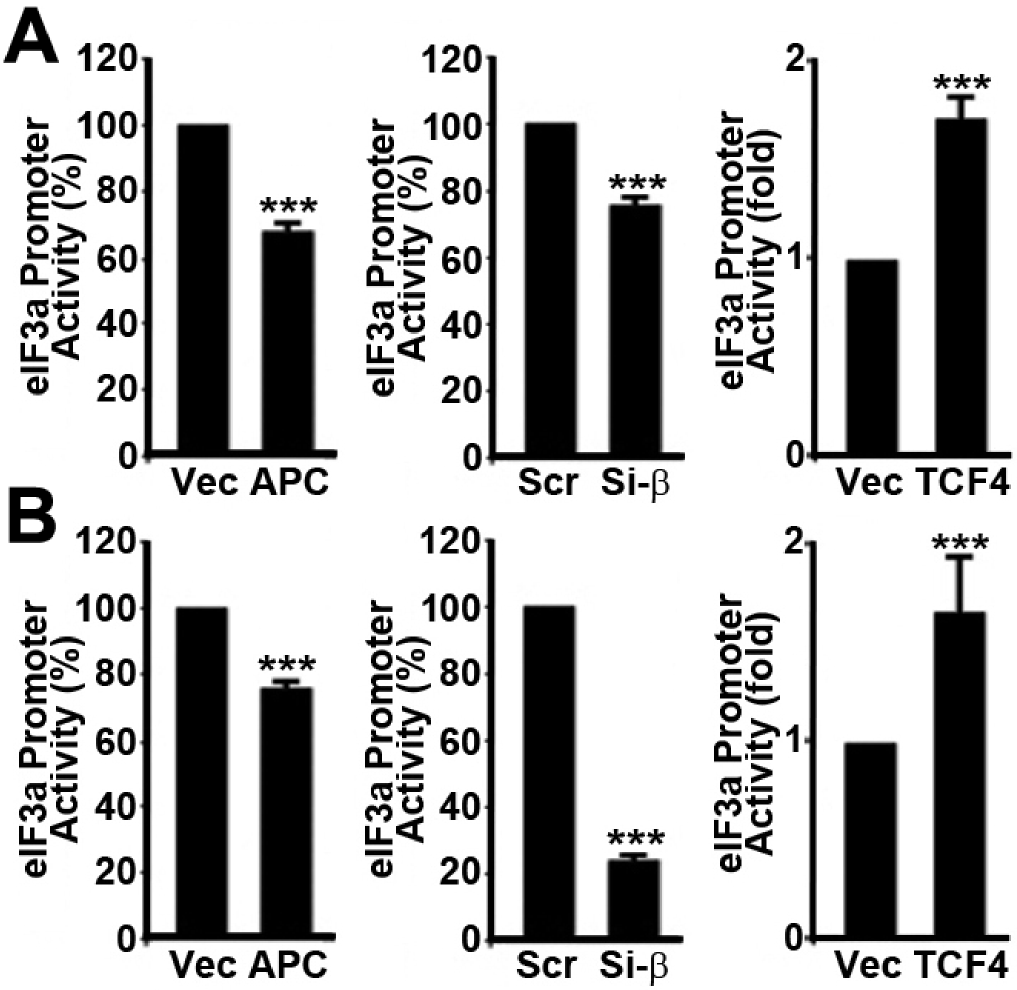

Figure S4. Regulation of eIF3a promoter activity by APC, β-catenin, and TCF4. Promoter activity of eIF3a in HEK293 (A) or H1299 (B) cells with over-expression of APC, TCF4 or with β-catenin knockdown as analyzed using reporter construct as described in Figure 5. Vector-transfected cells for APC and TCF4 over-expression or scrambled siRNA control for β-catenin knockdown were used as controls. (n=3, ***p<0.001).

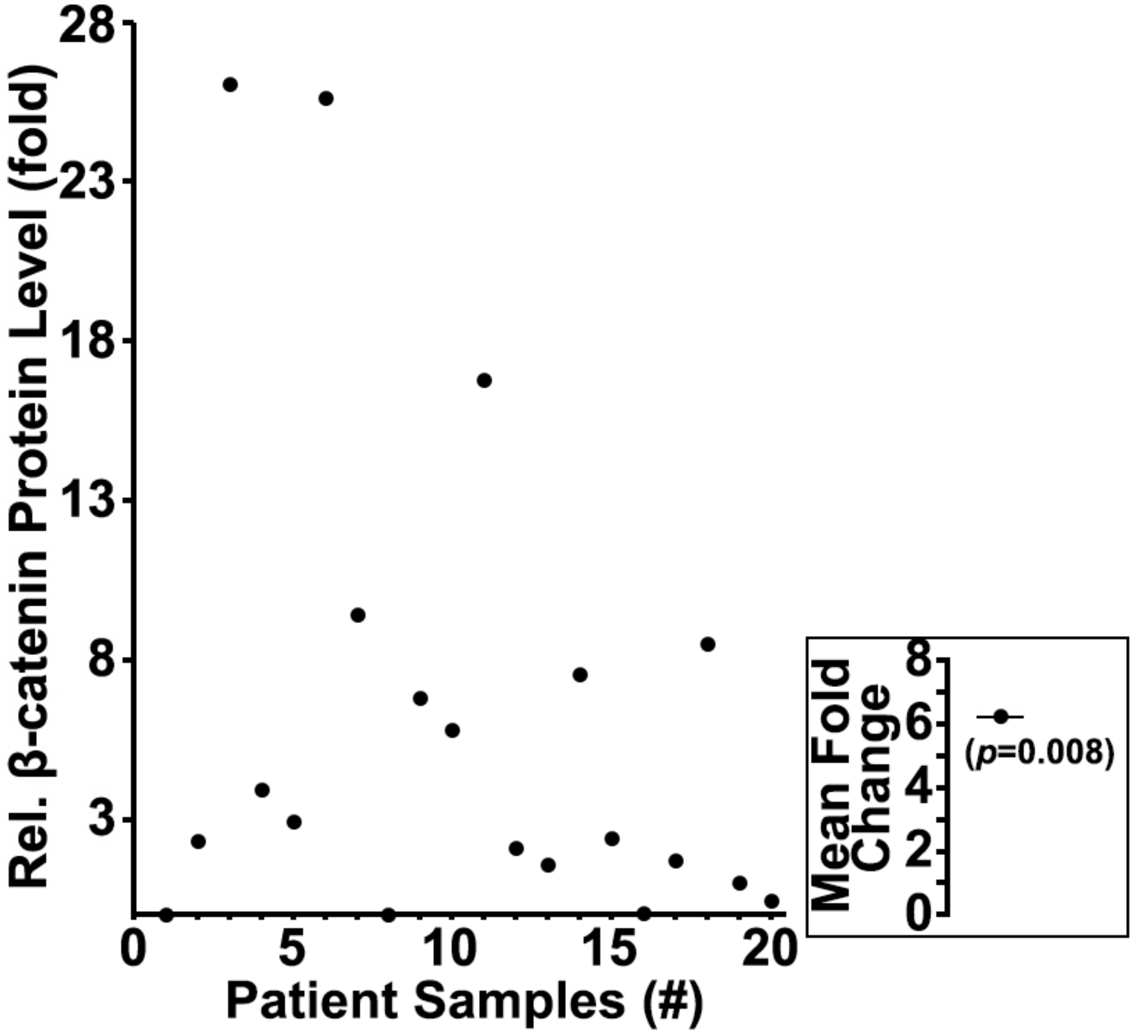

Figure S5. Fold change of β-catenin in cancer relative to paired normal tissues. Each dot represents fold change in the expression of β-catenin in each paired sample (cancer vs normal) as determined from the Western blot in Figure 6A. The mean fold change of β-catenin is shown in the box on the right.
